# Tackling the toxics in plastics packaging

**DOI:** 10.1371/journal.pbio.3000961

**Published:** 2021-03-30

**Authors:** Jane Muncke

**Affiliations:** Food Packaging Forum Foundation, Zurich, Switzerland

## Abstract

The widespread use of plastic packaging for storing, transporting, and conveniently preparing or serving foodstuffs is significantly contributing to the global plastic pollution crisis. This has led to many efforts directed toward amending plastic packaging’s end of life, such as recycling, or alternative material approaches, like increasingly using paper for food packaging. But these approaches often neglect the critical issue of chemical migration: When contacting foodstuffs, chemicals that are present in packaging transfer into food and thus unwittingly become part of the human diet. Hazardous chemicals, such as endocrine disrupters, carcinogens, or substances that bioaccumulate, are collectively referred to as “chemicals of concern.” They can transfer from plastic packaging into food, together with other unknown or toxicologically uncharacterized chemicals. This chemical transfer is scientifically undisputed and makes plastic packaging a known, and avoidable, source of human exposure to synthetic, hazardous, and untested chemicals. Here, I discuss this issue and highlight aspects in need of improvement, namely the way that chemicals present in food packaging are assessed for toxicity. Further, I provide an outlook on how chemical contamination from food packaging could be addressed in the future. Robust innovations must attempt systemic change and tackle the issue of plastic pollution and chemical migration in a way that integrates all existing knowledge.

## Abstract 2

The widespread use of plastic packaging for storing, transporting, and conveniently preparing or serving foodstuffs is significantly contributing to the global plastic pollution crisis. This Essay exhorts us to change the conversation about plastic packaging and address the chemicals that migrate into food.

## Plastics—The game changer

Plastic is an incredibly useful and fascinating material. During the last 60 years, it has contributed to economic prosperity and societal development. A Nobel Prize was awarded in 1963 to chemists for optimizing plastic polymerization [1]. And because plastic is abundant and inexpensive, it has enabled (mass-) consumption and access to affordable goods for most of humanity [2].

But there is also a dark side of plastic with its environmental persistence and the associated constant buildup of plastic pollution in the world’s oceans, sediments, and biota [3]. The fast moving good of plastic packaging makes up around 40% of global plastics production [4], and much of environmental plastics pollution stems from single-use plastic packaging items [5], notably plastic food packaging making up the most frequent type of beach waste [6].

Many different stakeholders, from civil society, academic research, governments, and industry, are now addressing the issue of plastic pollution, for example, by implementing improved waste management or banning certain single-use plastic items [7,8]. Indeed, plastics are not permanent materials and cannot be endlessly recycled because they lose their material properties during recycling [9]. This is one of the many challenges associated with plastics recycling in particular and addressing the plastics pollution challenge in general [10].

But that is not the only problem with plastic. In addition, plastics are not inert, and smaller molecules transfer from plastic packaging into food [11,12]. This process of chemical transfer is known as “migration,” and it has been shown also for hazardous chemicals and for chemicals with unknown toxicity [13,14]. This makes plastic packaging a relevant source of human exposure to synthetic chemicals.

### Chemicals in plastics: Known unknowns

**Fig 1:**
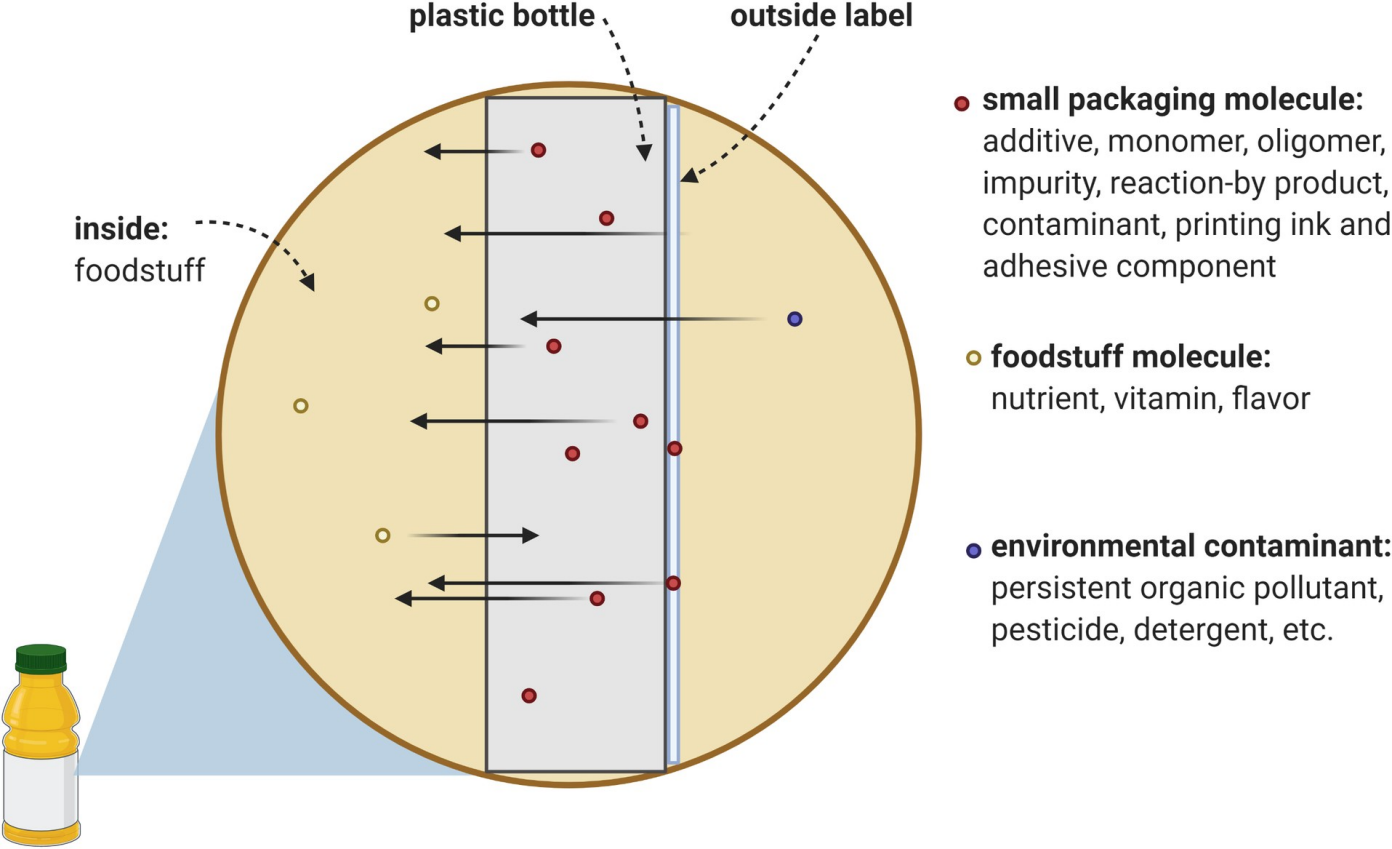
Schematic illustration of chemical migration from plastic food packaging into food. Small molecules that are present in the plastic packaging item (red dots) can transfer out of the plastic into the food. The same is true for small molecules present in the food (yellow dot), a process known as flavor scalping. Also, environmental contaminants (blue dots) can be absorbed in the plastic packaging and subsequently be released again, making this an issue for plastics recycling. Chemical migration, flavor scalping, and absorption depend on temperature, time, and the chemical properties of the packaging, the food and the chemicals that are transferring from one medium to another. Created with BioRender.com.

Indeed, there is growing apprehension about the hazardous chemicals used in the manufacture of plastics [12,15]. These chemicals have hazard properties such as being carcinogenic, mutagenic or reprotoxic, persistent, bioaccumulative and toxic, or endocrine disrupting. Several chemicals of concern are authorized for use in plastic food packaging [16], like bisphenol A (BPA; CAS 80-05-7) and di-(2-ethylhexyl) phthalate (DEHP; CAS 117-81-7). An overview of chemicals associated with plastic packaging (for food and nonfood uses) revealed that there are at least 148 substances with priority hazard properties for either human and/or environmental health [17]. This is worrying, because known or potentially hazardous chemicals contained in plastic can migrate into food, so the packaging that is used to protect food becomes a source of chemical contamination of the food [11] (Fig 1). Almost the entire human population therefore is very likely to be exposed to known hazardous chemicals from plastic food packaging. At the same time, the majority of the 4,283 chemicals identified to be (likely) used in plastic packaging manufacture lack toxicological data which precludes a hazard assessment [17]. This implies that there may be additional hazardous chemicals migrating from plastic packaging for which toxicity properties have yet to be determined.

But there is more: Apart from the substances that are intentionally used in the manufacture of plastics, all plastics also contain so-called non-intentionally added substances, or short NIAS, which have no technical function in the finished plastic material, but that are nevertheless present and can also migrate into food or the environment [18–20].

NIAS are a huge challenge. They can have various sources: impurities in the batches of monomers, the polymer’s building blocks; unwanted reaction by-products from polymerization; degradation products of additives, for example, antioxidants, that perform their function by degrading into different chemicals which then also are present in plastics; and contaminants present in recycled plastics [21,22]. In 2007, the UK’s Food Standards Agency studied “reaction and breakdown products from starting substances used to produce food contact plastics” in 6 different, commonly used plastics types [23]. Their conclusion: “A large number of substances remain either unidentified or with an ambiguous identification only.” And even though this study was conducted over 10 years ago and analytical techniques have improved somewhat since, there are still unknown chemicals present in and migrating from plastics [24–26]. This means that some of the chemicals migrating from plastic packaging into food (and the environment, when plastic packaging becomes a pollutant) remain uncharacterized.

### Changing the game on plastics packaging: Talking toxics

On the other hand, several well-known chemicals of concern are used to make plastics and migrate from plastics, for example, BPA, DEHP, and melamine (CAS 108-78-1) [27,28]. Other hazardous chemicals are present as NIAS, like nonylphenol (various CAS, e.g., 84852-15-3), a breakdown product of an antioxidant [29]. These substances are studied widely, both for their migration from plastic and their toxicity properties [54]. Public awareness for their hazardousness has led to some substitution with other chemicals, but unfortunately, in some cases, to replacement with equally toxic substances, like bisphenol S (CAS 80-09-1) or bisphenol F (CAS 620-92-8) [30]. This type of change is best described as a “Whack-a-Mole” approach which solves one problem only to create another—not a sustainable way forward.

**Fig 2:**
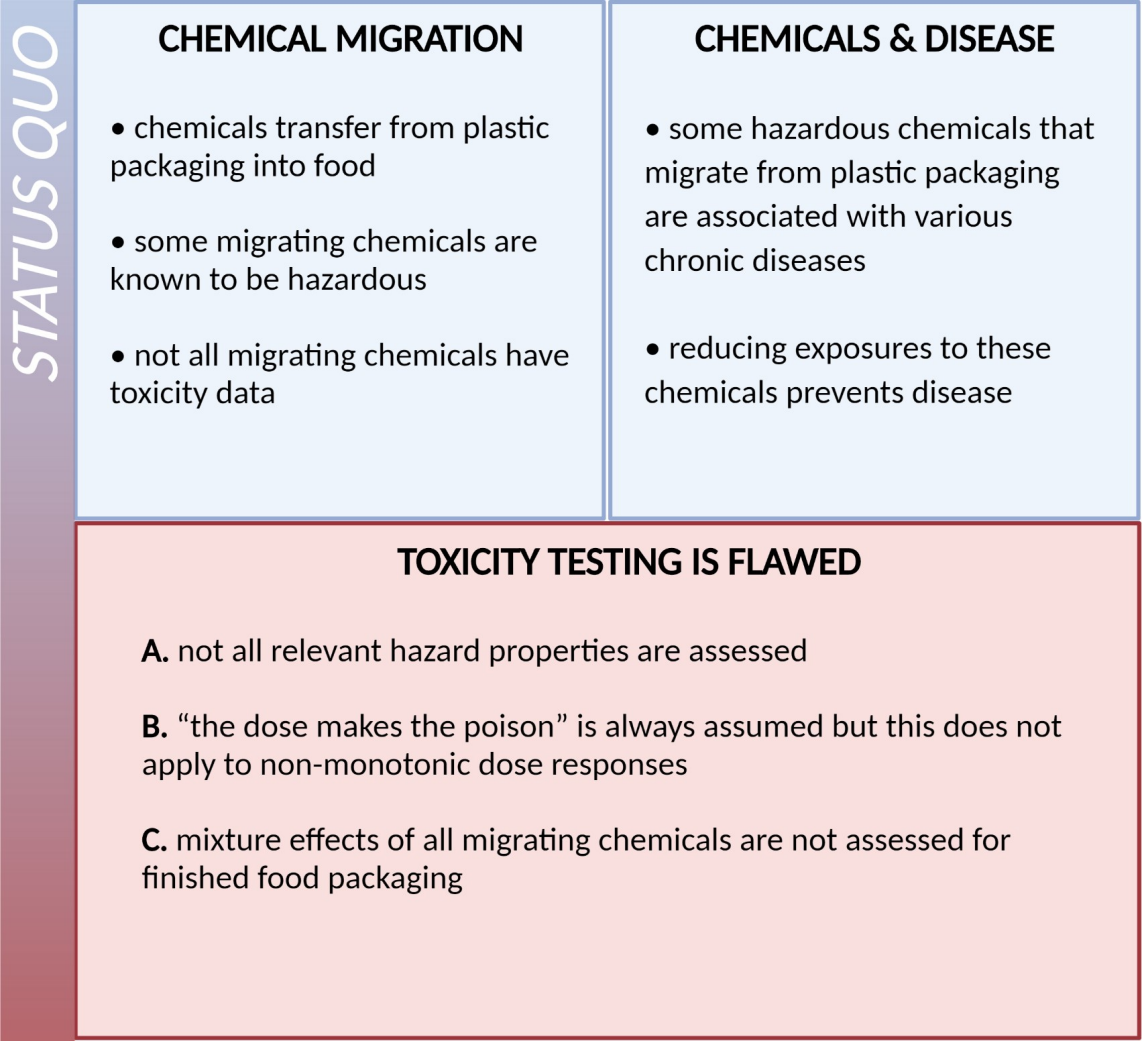
Chemical migration from packaging into food—the status quo. Chemicals migrate from plastic packaging into food, and some of the migrating chemicals are known to be hazardous. Chronic exposure to certain hazardous chemicals is associated with avoidable chronic disease. This means that reducing exposure to hazardous chemicals from plastic food packaging contributes to disease prevention. Unknown or untested chemicals that migrate into food must also be addressed, as they may include hazardous chemicals.

**Fig 3:**
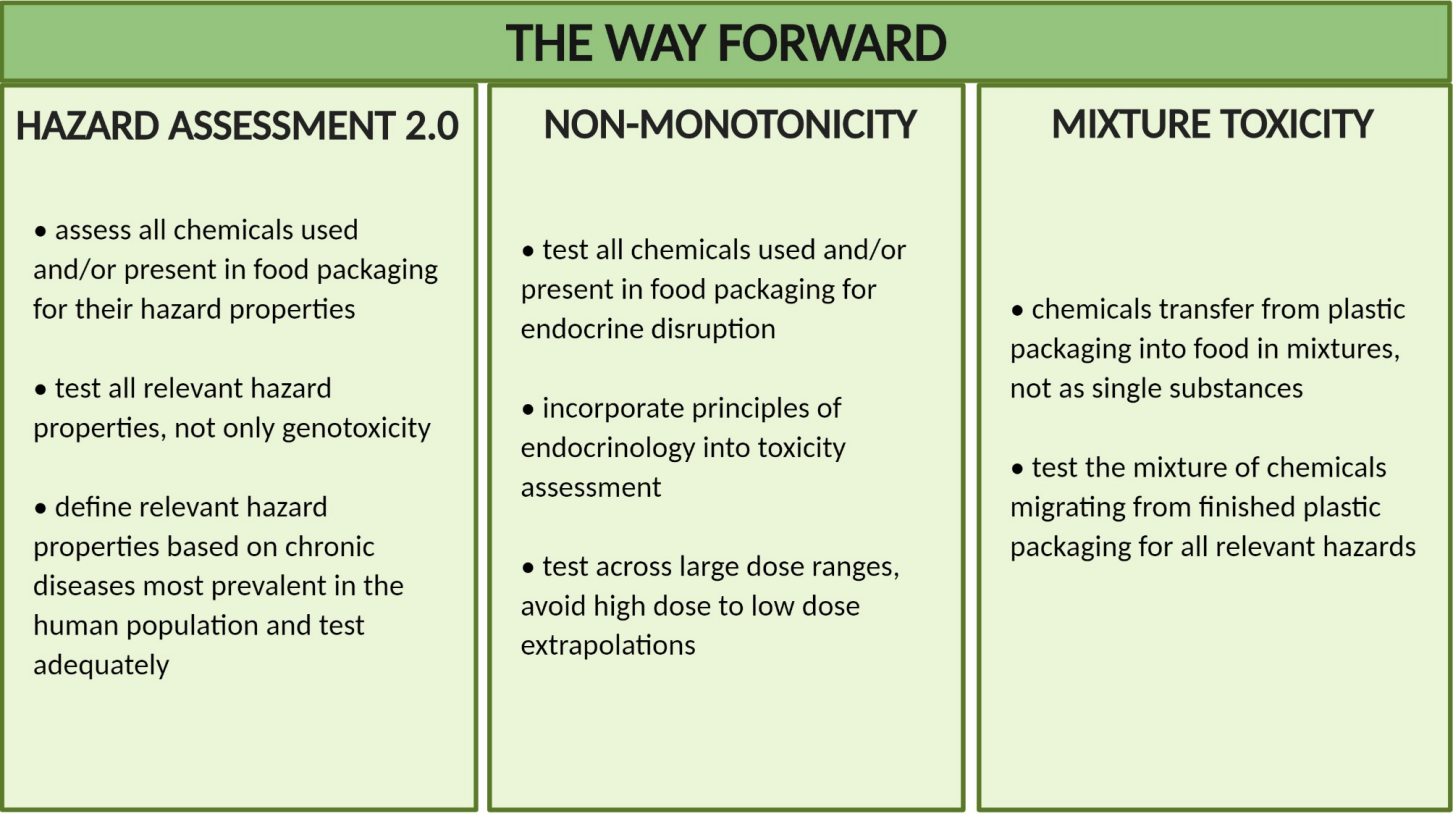
The 3 key areas for improving the chemical safety of food packaging, based on current knowledge: (A) expanding hazard assessment to include other relevant toxicities; (B) systematically assessing migrating chemicals for their ability to disrupt the endocrine system by using appropriate testing that allow for identification of non-monotonic dose responses; (C) tackling mixture toxicity to identify toxic effects of all chemicals migrating simultaneously.

Therefore, I argue that the current approach to understanding and improving the chemical safety of plastics food packaging needs to be revised (Fig 2). Specifically, 3 key aspects need to be considered (Fig 3): (1) expanding the scope of toxicity testing beyond genotoxicity; (2) addressing non-monotonic dose responses in chemical risk assessment; and (3) finding practical solutions to assessing and managing mixture toxicity.

Each of these aspects is challenging, but some first approaches for dealing with them are promising.

### Preventing chronic disease: Weeding out the toxics

Firstly, current safety assessments for chemicals used in and migrating from plastic food packaging are narrowly focused on genotoxicity as hazard property. This is meaningful, because genotoxic chemicals are implicated in the causation of cancers, and cancers are highly prevalent in the human population. Weeding out genotoxic chemicals from food packaging is critical because it supports cancer prevention, and prevention is the key to battling this cruel disease [31].

By the same logic, it is reasonable and based on current scientific knowledge to also exclude chemicals with other hazard properties that are associated with highly prevalent, noncancerous chronic diseases [54]. Notably, cardiovascular disease is the most relevant cause for premature death [32], and for some chemicals, there is evidence that they affect cardiac function—for example, the plastic chemical BPA [33]. Similarly, metabolic diseases are increasing globally, and several obesogenic or metabolic disrupting chemicals are known to be associated with plastics [34]. Therefore, chemical hazard assessment for plastic substances needs to be expanded beyond the current genotoxicity focus to address additional hazard properties of relevance to human health such as endocrine disruption, neurotoxicity, and immunotoxicity, to improve the prevention of chronic diseases that are affected by certain hazardous chemical exposures.

One promising approach is to apply the key characteristics concept. Developed by scientists at the International Agency for Research on Cancer, key characteristics of carcinogens describe properties of chemicals that are known to cause cancer in humans [35]. These key characteristics can then be used in the screening of other chemicals, to identify if they possess these sets of properties that make them likely human carcinogens [36]. Importantly, key characteristics (for example, if a chemical induces oxidative stress) can be determined using appropriate in vitro assays, so identifying a chemical’s hazard properties would not require extensive animal tests. Key characteristics have also now been developed for endocrine disrupting chemicals [37], male reproductive toxicants [38], and female reproductive toxicants [39], and currently, work is ongoing to tackle the key characteristics for cardiotoxicants and immunotoxicants, chemicals that adversely impact the immune system, which is particularly important during the Coronavirus Disease 2019 (COVID-19) pandemic [40]. In conclusion, the key characteristics could be a first stepping-stone on the path to a more complete hazard characterization of chemicals migrating from plastic food packaging. Further work is necessary before this concept becomes applicable in the regulatory toxicology context, for example, identifying the appropriate in vitro bioassays to use [41].

### When less is more: Non-monotonic dose responses and endocrine disrupting chemicals

In 1564, the medieval scholar Philippus Bombastus von Hohenheim, known as Paracelsus, wrote “What is there that is not poison? All things are poison and nothing (is) without poison. Solely the dose determines that a thing is not a poison” [42]. This has been famously misquoted and morphed into the central paradigm of toxicology: “the dose makes the poison,” implying that with increasing concentration of chemical exposure, the risk of adverse health impacts is assumed to increase. If this paradigm’s logic is reverted, it can be interpreted to mean that low levels of exposures to (synthetic, untested) chemicals are of negligible risk. As a matter of fact, this is currently the underlying assumption in chemical risk assessment for plastic’s ingredients that transfer from packaging into food—at low levels, the effects of exposures to untested chemicals are assumed to be insignificant [14].

But this assumption is not always valid. Indeed, chemicals that interfere with the endocrine system, so-called endocrine disrupting chemicals, can display non-monotonic dose response relationships where effects can be seen at lower doses that are not observed at higher doses. As consequence, these types of dose responses defy toxicology’s paradigm because also low, “negligible” doses can lead to adverse effects [43]. So, while non-monotonic dose responses are common in nature and, therefore, have to be expected to also occur with synthetic chemicals, it has been an uphill battle to get recognition for this biological phenomenon in the regulatory toxicology community, leading to subsequent regulatory action [44–47]. Still, there are many examples of chemicals in plastic packaging that are known or suspected endocrine disrupting chemicals [26,48–53], such as benzophenone (CAS 119-61-9), 2,4-di-tert-butylphenol (CAS 96-76-4), or the styrene dimer 1,3-diphenylpropane (CAS 1081-75-0). Endocrine disruption is currently not routinely being addressed when chemicals are authorized for use in (plastic) food packaging [14,54].

Low-dose effects are especially concerning if they occur during early life, like during fetal development and perinatally [55]. Such chemical exposures can adversely affect the quality of life for the next generation, making it a sustainability topic and a potential generational conflict issue [56]. Therefore, there is a moral obligation for regulators to reduce harm by addressing this issue, for example, by dealing with the presence of endocrine disrupting chemicals in plastic food packaging in a science-based and systematic way [57,58].

### Something from nothing: Facing the reality of mixture toxicity

In chemical risk assessment for plastic food packaging, substances are evaluated individually, and safe exposure limits are set substance by substance. However, chemicals migrate in mixtures, and the human population is not exposed to only 1 (synthetic) chemical at a time. This implies that human exposure is far more complex than currently addressed by chemical risk assessment approaches. Indeed, adverse effects of chemical exposures can occur “from nothing”: Combinations of chemicals that are all present at or below their individual effect thresholds, or safe exposure limits, have been shown to cause effects when present in mixtures [59].

A possible way forward is offered by the use of bioassays, where all the chemicals migrating from plastic into food could be assessed as a “cocktail” [60,61], and regulators prioritize mixtures of concern based on effect-based thresholds, as has been proposed for assessments of surface waters [62]. Another approach is the use of an uncertainty factor for mixtures (“mixture assessment factor” (MAF)): In the absence of detailed information, the overall acceptable exposure to plastic chemicals would be lowered by the MAF [63]. For example, the legally acceptable levels of known and unknown chemicals migrating into food would be reduced by applying the MAF. A similar approach can be used for plastics in the environment, when plastic packaging becomes a pollutant.

### Solutions for tomorrow: Taking a holistic approach

**Fig 4:**
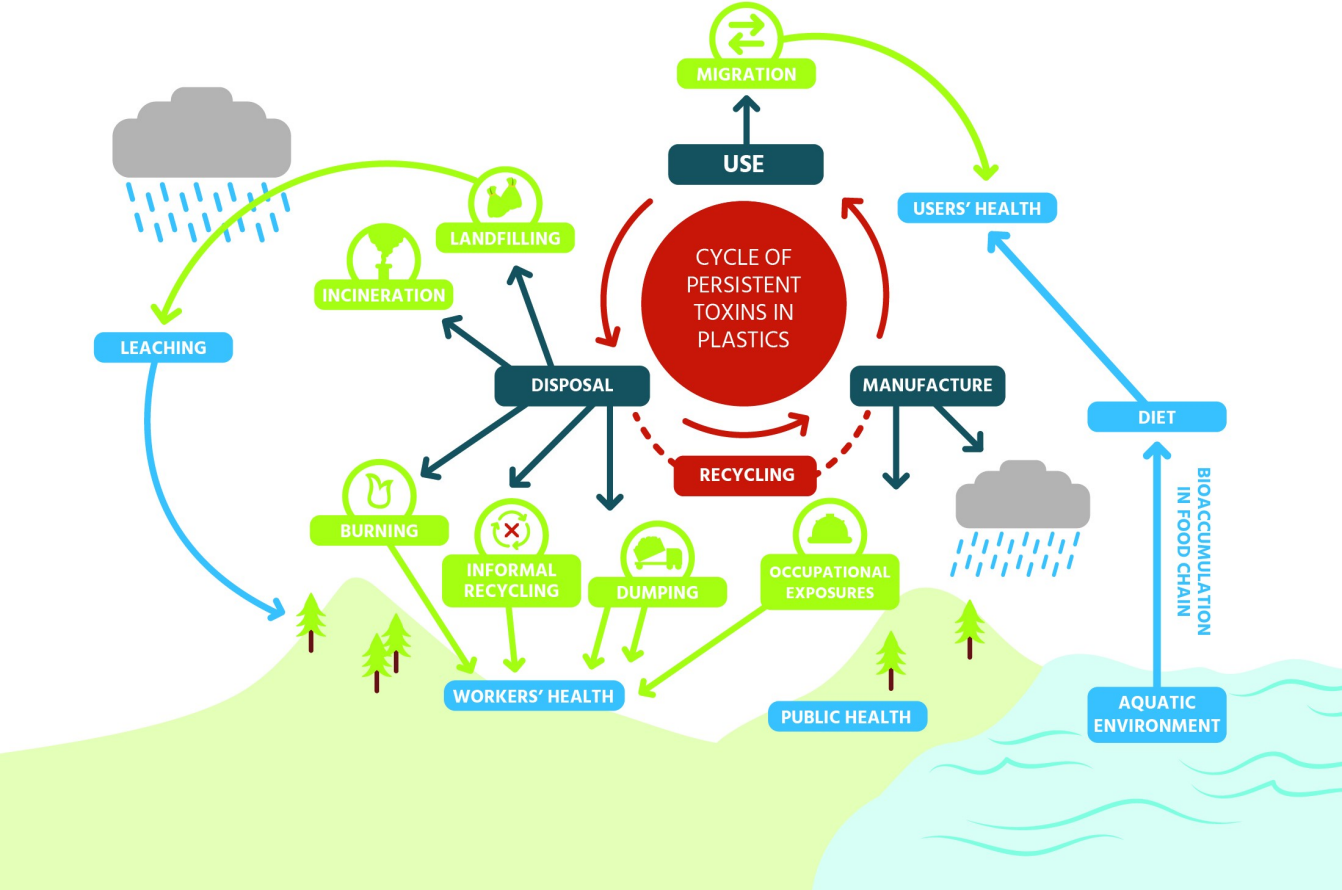
Schematic overview of the human and environmental health impacts of the plastics life cycle. Adapted and reprinted with permission from the Health and Environment Alliance [67].

**Fig 5:**
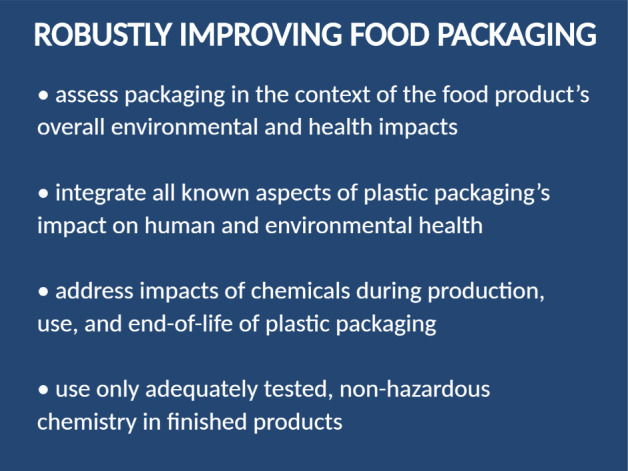
Robustly improving food packaging requires consideration of numerous aspects to achieve systemic change that does not result in the creation of new wicked problems.

Tackling plastic pollution is by far not an easy or simple task. In our globalized food system that depends on centralized food processing and includes many economy-of-scale business models, the use of plastic packaging is currently essential to enable transportation, extended shelf life, and convenience of food products while being economically profitable. At the same time, plastics in contact with food are a relevant source of human exposure to synthetic chemicals. And when plastic packaging becomes plastic pollution, chemicals and microplastics also transfer into the environment—and vice versa: Persistent organic pollutants (POPs) such as dichlorodiphenyltrichloroethane (DDT) or polychlorinated biphenyls (PCBs) are absorbed by marine (micro-) plastics [64]. Many plastic chemicals of relevance for human and environmental exposure are toxicologically not characterized, and some are unknown—a knowledge gap that can be considered inacceptable. Improving our society’s use of plastic requires sorting out the toxics in plastic food packaging as one of the necessary first steps to reduce the unwanted and preventable effects of this useful material. But when ameliorating the issue of plastic packaging, it is also essential that “regrettable substitutions” are avoided, meaning that plastic packaging must not be substituted with alternative materials containing hazardous and/or untested chemicals. An example is paper packaging that can be of as great concern for leaching hazardous chemicals into food as plastic. By the same token, another regrettable substitution is the recycling of plastics that contain hazardous chemicals. In short, attempts to make plastic packaging—or its replacements—more benign to human and environmental health must address hazardous chemicals as one of the key issues, across the entire life cycle of plastics (Fig 4). The plastic pollution problem requires systemic thinking that shies away from quick fixes addressing only 1 symptom of the larger problem [65]. Instead, robust innovations will be built on a thorough, holistic understanding of the plastics problem that must be developed by integrating all available knowledge—including hazardous chemicals—across plastic’s entire life cycle [66] (Fig 5).

## Abbreviations

BPA: bisphenol A
COVID-19: Coronavirus Disease 2019
DDT: dichlorodiphenyltrichloroethane
DEHP: di-(2-ethylhexyl) phthalate
MAF: mixture assessment factor
NIAS: non-intentionally added substances
PCB: polychlorinated biphenyl
POP: persistent organic pollutant

## References

[1] The Nobel Prize. The Nobel Prize in Chemistry 1963: converting catalysts NobelPrize.org: Nobel Media AB. 2009. Available from: https://www.nobelprize.org/prizes/chemistry/1963/speedread/.

[2] Freinkel S. Plastic: A toxic love story. Boston, New York: Houghton Mifflin Harcourt; 2011.

[3] Beaumont NJ, Aanesen M, Austen MC, Börger T, Clark JR, Cole M, et al. Global ecological, social and economic impacts of marine plastic. Mar Pollut Bull. 2019;142:189–95. 10.1016/j.marpolbul.2019.03.022 31232294

[4] Plastics Europe. Plastics—The facts. An analysis of European plastics production, demand and waste data.: Plastics Europe; October 2019.

[5] Ocean Conservancy. Building A Swell. 2018 report. Washington D.C.: Ocean Conservancy; 2018.

[6] Parker L. Plastic food packaging was most common beach trash in 2018. Natl Geogr. 9 2019;2019:3.

[7] World Economic Forum. Global Plastic Action Partnership. 2018. Available from: https://globalplasticaction.org/.

[8] Council adopts ban on single-use plastics [press release]. Council of the European Union. 2019;21:2019.

[9] Conte F, Dinkel F, Kägi T, Heim T. Permanent materials. Scientific background Basel: Carbotech. 2014.

[10] Dauvergne P. Why is the global governance of plastic failing the oceans? Glob Environ Chang. 2018;51:22–31.

[11] Muncke J, Andersson A-M, Backhaus T, Boucher JM, Carney Almroth B, Castillo Castillo A, et al. Impacts of food contact chemicals on human health: a consensus statement. Environ Health. 2020;19(1):25. 10.1186/s12940-020-0572-5 32122363 PMC7053054

[12] Hahladakis JN, Velis CA, Weber R, Iacovidou E, Purnell P. An overview of chemical additives present in plastics: Migration, release, fate and environmental impact during their use, disposal and recycling. J Hazard Mater. 2018;344:179–99. 10.1016/j.jhazmat.2017.10.014 29035713

[13] Geueke B, Muncke J. Substances of very high concern in food contact materials: Migration and regulatory background. Packag Technol Sci. 2018;31(12):757–69.

[14] Muncke J, Backhaus T, Geueke B, Maffini MV, Martin OV, Myers JP, et al. Scientific challenges in the risk assessment of food contact materials. Environ Health Perspect. 2017;125(9):095001. 10.1289/EHP644 28893723 PMC5915200

[15] Halden RU. Plastics and Health Risks. Annu Rev Public Health. 2010;31:178–94. 10.1146/annurev.publhealth.012809.103714 20070188

[16] Geueke B, Wagner CC, Muncke J. Food contact substances and chemicals of concern: a comparison of inventories. Food Addit Contam Part A Chem Anal Control Expo Risk Assess. 2014;31(8):1438–50. 10.1080/19440049.2014.931600 24999917

[17] Groh KJ, Backhaus T, Carney-Almroth B, Geueke B, Inostroza PA, Lennquist A, et al. Overview of known plastic packaging-associated chemicals and their hazards. Sci Total Environ. 2019;651:3253–68. 10.1016/j.scitotenv.2018.10.015 30463173

[18] Nerin C, Alfaro P, Aznar M, Domeño C. The challenge of identifying non-intentionally added substances from food packaging materials: A review. Anal Chim Acta. 2013;775(2 May 2013):14–24. 10.1016/j.aca.2013.02.028 23601971

[19] Geueke B. Non-intentionally added substances (NIAS). Food Packaging Forum Foundation. 2018 6;2018. Available from: https://www.foodpackagingforum.org/food-packaging-health/non-intentionally-added-substances-nias

[20] Wrona M, Nerín C. Analytical Approaches for Analysis of Safety of Modern Food Packaging: A Review. Molecules. 2020;25(3):752. 10.3390/molecules25030752 32050512 PMC7037176

[21] Horodytska O, Cabanes A, Fullana A. Non-intentionally added substances (NIAS) in recycled plastics. Chemosphere. 2020;251:126373. 10.1016/j.chemosphere.2020.126373 32163780

[22] Dreolin N, Aznar M, Moret S, Nerin C. Development and validation of a LC–MS/MS method for the analysis of bisphenol a in polyethylene terephthalate. Food Chem. 2019;274:246–53. 10.1016/j.foodchem.2018.08.109 30372934

[23] Bradley E, Coulier L. An investigation into the reaction and breakdown products from starting substances used to produce food contact plastics. Report. London: Central Science Laboratory; 2007 8 2007. Report No.: FD 07/01 Contract No.: FD07/01. Available from: https://webarchive.nationalarchives.gov.uk/20100816210544/http://www.foodbase.org.uk/results.php?f_report_id=518

[24] García Ibarra V, Rodríguez Bernaldo de Quirós A, Paseiro Losada P, Sendón R. Non-target analysis of intentionally and non intentionally added substances from plastic packaging materials and their migration into food simulants. Food Packag Shelf Life. 2019;21:100325.

[25] Martínez-Bueno MJ, Gómez Ramos MJ, Bauer A, Fernández-Alba AR. An overview of non-targeted screening strategies based on high resolution accurate mass spectrometry for the identification of migrants coming from plastic food packaging materials. TrAC Trends Anal Chem. 2019;110:191–203.

[26] Zimmermann L, Dierkes G, Ternes TA, Völker C, Wagner M. Benchmarking the in Vitro Toxicity and Chemical Composition of Plastic Consumer Products. Environ Sci Technol. 2019;53(19):11467–77. 10.1021/acs.est.9b02293 31380625

[27] Mannoni V, Padula G, Panico O, Maggio A, Arena C, Milana M-R. Migration of formaldehyde and melamine from melaware and other amino resin tableware in real life service. Food Addit Contam Part A Chem Anal Control Expo Risk Assess. 2017;34(1):113–25. 10.1080/19440049.2016.1252467 27824529

[28] Fasano E, Bono-Blay F, Cirillo T, Montuori P, Lacorte S. Migration of phthalates, alkylphenols, bisphenol A and di(2-ethylhexyl)adipate from food packaging. Food Control. 2012;27(1):132–8.

[29] Hamlin HJ, Marciano K, Downs CA. Migration of nonylphenol from food-grade plastic is toxic to the coral reef fish species Pseudochromis fridmani. Chemosphere. 2015;139(0):223–8. 10.1016/j.chemosphere.2015.06.032 26134675

[30] CHEM Trust. From BPA to BPZ: a toxic soup? How companies switch from a known hazardous chemical to one with similar properties, and how regulators could stop them. CHEM Trust,; 2018.

[31] Madia F, Worth A, Whelan M, Corvi R. Carcinogenicity assessment: Addressing the challenges of cancer and chemicals in the environment. Environ Int. 2019;128:417–29. 10.1016/j.envint.2019.04.067 31078876 PMC6520474

[32] WHO. Noncommunicable diseases. Fact sheet. 2018. Available from: http://www.who.int/mediacentre/factsheets/fs355/en/.

[33] Yan S, Chen Y, Dong M, Song W, Belcher SM, Wang H-S. Bisphenol A and 17β-Estradiol Promote Arrhythmia in the Female Heart via Alteration of Calcium Handling. PLoS ONE. 2011;6(9):e25455. 10.1371/journal.pone.0025455 21980463 PMC3181279

[34] Heindel JJ, Blumberg B. Environmental Obesogens: Mechanisms and Controversies. Annu Rev Pharmacol Toxicol. 2019;59:89–106. 10.1146/annurev-pharmtox-010818-021304 30044726 PMC6559802

[35] Smith MT, Guyton KZ, Gibbons CF, Fritz JM, Portier CJ, Rusyn I, et al. Key Characteristics of Carcinogens as a Basis for Organizing Data on Mechanisms of Carcinogenesis. Environ Health Perspect. 2016;124(6):713–21. 10.1289/ehp.1509912 26600562 PMC4892922

[36] Guyton KZ, Rusyn I, Chiu WA, Corpet DE, van den Berg M, Ross MK, et al. Application of the key characteristics of carcinogens in cancer hazard identification. Carcinogenesis. 2018;39(4):614–22. 10.1093/carcin/bgy031 29562322 PMC5888955

[37] La Merrill MA, Vandenberg LN, Smith MT, Goodson W, Browne P, Patisaul HB, et al. Consensus on the key characteristics of endocrine-disrupting chemicals as a basis for hazard identification. Nat Rev Endocrinol. 2020;16(1):45–57. 10.1038/s41574-019-0273-8 31719706 PMC6902641

[38] Arzuaga X, Smith MT, Gibbons CF, Skakkebaek NE, Yost EE, Beverly BEJ, et al. Proposed Key Characteristics of Male Reproductive Toxicants as an Approach for Organizing and Evaluating Mechanistic Evidence in Human Health Hazard Assessments. Environ Health Perspect. 2019;127(6):65001. 10.1289/EHP5045 31199676 PMC6792367

[39] Luderer U, Eskenazi B, Hauser R, Korach KS, McHale CM, Moran F, et al. Proposed Key Characteristics of Female Reproductive Toxicants as an Approach for Organizing and Evaluating Mechanistic Data in Hazard Assessment. Environ Health Perspect. 2019;127(7):075001. 10.1289/EHP4971 31322437 PMC6791466

[40] Smith M. The key characteristics (KC) approach to hazard identification [video]. Food Packaging Forum; 2020 [webinar]. Available from: https://www.foodpackagingforum.org/events/key-characteristics-for-identifying-hazardous-chemicals.

[41] Smith MT, Guyton KZ, Kleinstreuer N, Borrel A, Cardenas A, Chiu WA, et al. The Key Characteristics of Carcinogens: Relationship to the Hallmarks of Cancer, Relevant Biomarkers, and Assays to Measure Them. Cancer Epidemiol Biomarkers Prev. 2020. 10.1158/1055-9965.EPI-19-1346 PMC7483401 32152214

[42] Deichmann WB, Henschler D, Holmsted B, Keil G. What is there that is not poison? A study of the Third Defense by Paracelsus. Arch Toxicol. 1986;58(4):207–13. 10.1007/BF00297107 3521542

[43] Vandenberg LN, Colborn T, Hayes TB, Heindel JJ, Jacobs DR, Lee D-H, et al. Hormones and Endocrine-Disrupting Chemicals: Low-Dose Effects and Nonmonotonic Dose Responses. Endocr Rev. 2012;33(3):378–455. 10.1210/er.2011-1050 22419778 PMC3365860

[44] Beausoleil C, Beronius A, Bodin L, Bokkers BGH, Boon PE, Burger M, et al. Review of non-monotonic dose-responses of substances for human risk assessment. EFSA Supporting Publications. 2016;13(5):1027E.

[45] Vandenberg LN, Prins GS, Patisaul HB, Zoeller RT. The Use and Misuse of Historical Controls in Regulatory Toxicology: Lessons from the CLARITY-BPA Study. Endocrinology. 2020;161(5):17. 10.1210/endocr/bqz014 31690949 PMC7182062

[46] Vandenberg LN, Hunt PA, Gore AC. Endocrine disruptors and the future of toxicology testing—lessons from CLARITY–BPA. Nat Rev Endocrinol. 2019;15(6):366–74. 10.1038/s41574-019-0173-y 30842650

[47] Kassotis CD, Vandenberg LN, Demeneix BA, Porta M, Slama R, Trasande L. Endocrine-disrupting chemicals: economic, regulatory, and policy implications. Lancet Diabetes Endocrinol. 2020;8(8):719–30. 10.1016/S2213-8587(20)30128-5 32707119 PMC7437819

[48] Muncke J. Exposure to endocrine disrupting compounds via the food chain: Is packaging a relevant source? Sci Total Environ. 2009;407(16):4549–59. 10.1016/j.scitotenv.2009.05.006 19482336

[49] Bergmann AJ, Simon E, Schifferli A, Schönborn A, Vermeirssen ELM. Estrogenic activity of food contact materials—evaluation of 20 chemicals using a yeast estrogen screen on HPTLC or 96-well plates. Anal Bioanal Chem. 2020. 10.1007/s00216-020-02701-w 32458016 PMC7329773

[50] Mertl J, Kirchnawy C, Osorio V, Grininger A, Richter A, Bergmair J, et al. Characterization of Estrogen and Androgen Activity of Food Contact Materials by Different In Vitro Bioassays (YES, YAS, ERalpha and AR CALUX) and Chromatographic Analysis (GC-MS, HPLC-MS). PLoS ONE. 2014;9(7):e100952. 10.1371/journal.pone.0100952 25000404 PMC4085075

[51] Kirchnawy C, Mertl J, Osorio V, Hausensteiner H, Washüttl M, Bergmair J, et al. Detection and Identification of Oestrogen-Active Substances in Plastic Food Packaging Migrates. Packag Technol Sci. 2014;27(6):467–78.

[52] Wagner M, Oehlmann J. Endocrine disruptors in bottled mineral water: Estrogenic activity in the E-Screen. J Steroid Biochem Mol Biol. 2011;127(1):128–35. 10.1016/j.jsbmb.2010.10.007 21050888

[53] Wagner M, Oehlmann J. Endocrine disruptors in bottled mineral water: total estrogenic burden and migration from plastic bottles. Environ Sci Pollut Res. 2009;16(3):278–86. 10.1007/s11356-009-0107-7 19274472

[54] Kahn LG, Philippat C, Nakayama SF, Slama R, Trasande L. Endocrine-disrupting chemicals: implications for human health. Lancet Diabetes Endocrinol. 2020;8(8):703–18. 10.1016/S2213-8587(20)30129-7 32707118 PMC7437820

[55] Balbus JM, Barouki R, Birnbaum LS, Etzel RA, Gluckman PD, Grandjean P, et al. Early-life prevention of non-communicable diseases. Lancet. 2013;381(9860):3–4. 10.1016/S0140-6736(12)61609-2 23290956 PMC3849695

[56] Grandjean P, Barouki R, Bellinger DC, Casteleyn L, Chadwick LH, Cordier S, et al. Life-Long Implications of Developmental Exposure to Environmental Stressors: New Perspectives. Endocrinology. 2015;156(10):3408–15. 10.1210/EN.2015-1350 26241067 PMC4588822

[57] National Research Council. Review of the Environmental Protection Agency’s State-of-the-Science evaluation of nonmonotonic dose-response relationships as they apply to endocrine disruptors. Washington, DC: The National Academies Press; 2014.

[58] Hass U, Christiansen S, Andersson A-M, Holbech H, Bjerregaard P. Report on interpretation of knowledge on endocrine disrupting substances (EDs)—what is the risk?; 2019. Available from: https://www.food.dtu.dk/english/news/2019/02/do-risk-assessments-of-endocrine-disruptors-provide-sufficient-protection

[59] Kortenkamp A, Faust M. Regulate to reduce chemical mixture risk. Science. 2018;361(6399):224–6. 10.1126/science.aat9219 30026211

[60] Groh KJ, Muncke J. In Vitro Toxicity Testing of Food Contact Materials: State-of-the-Art and Future Challenges. Compr Rev Food Sci Food Saf. 2017;16(5):1123–50. 10.1111/1541-4337.12280 33371616

[61] Severin I, Souton E, Dahbi L, Chagnon MC. Use of bioassays to assess hazard of food contact material extracts: State of the art. Food Chem Toxicol. 2017;105:429–47. 10.1016/j.fct.2017.04.046 28476634

[62] Escher BI, Aїt-Aїssa S, Behnisch PA, Brack W, Brion F, Brouwer A, et al. Effect-based trigger values for in vitro and in vivo bioassays performed on surface water extracts supporting the environmental quality standards (EQS) of the European Water Framework Directive. Sci Total Environ. 2018;628–629:748–65. 10.1016/j.scitotenv.2018.01.340 29454215

[63] Drakvik E, Altenburger R, Aoki Y, Backhaus T, Bahadori T, Barouki R, et al. Statement on advancing the assessment of chemical mixtures and their risks for human health and the environment. Environ Int. 2020;105267:134. 10.1016/j.envint.2019.105267 31704565 PMC6979318

[64] Carbery M, O’Connor W, Palanisami T. Trophic transfer of microplastics and mixed contaminants in the marine food web and implications for human health. Environ Int. 2018;115:400–9. 10.1016/j.envint.2018.03.007 29653694

[65] Lau WWY, Shiran Y, Bailey RM, Cook E, Stuchtey MR, Koskella J, et al. Evaluating scenarios toward zero plastic pollution. Science. 2020:eaba9475. 10.1126/science.aba9475 32703909

[66] Leslie HA, Leonards PEG, Brandsma SH, de Boer J, Jonkers N. Propelling plastics into the circular economy—weeding out the toxics first. Environ Int 2016;94:230–4. 10.1016/j.envint.2016.05.012 27262786

[67] Health and Environment Alliance. Turning the Plastic Tide–The chemicals and plastics that put our health at risk. HEAL; 2020. Available from: https://www.env-health.org/turning-the-plastic-tide-the-chemicals-in-plastic-that-put-our-health-at-risk/

